# Physico-Chemical and Microbiological Differences between Mains and Bottled Water, in an Area in the Central Area of Romania

**DOI:** 10.3390/ijerph20021115

**Published:** 2023-01-08

**Authors:** Ruta Florina, Avram Calin, Maior Raluca

**Affiliations:** 1 Department Community Nutrition and Food Safety, George Emil Palade University of Medicine, Pharmacy, Science and Technology, 540142 Targu Mures, Romania; 2 Department Medical Informatics and Biostatistics, George Emil Palade University of Medicine, Pharmacy, Science and Technology, 540142 Targu Mures, Romania; 3 Nutricare Clinic, 540342 Targu Mures, Romania

**Keywords:** water, bottled water, mineral water, microbiological determinations

## Abstract

The human body is mostly made up of water. The largest proportion of the human body is water, hence the growing interest of specialists in defining the importance of water in the body and the risks of dehydration. This study determined the physico-chemical and microbiological parameters of the drinking water distributed in the public network in the area of operation of Water Distribution Operator in Mureș County and samples of bottled water existing on the market in Mureş County (mineral, spring or table). The water supplied to the network can be safely consumed. This is demonstrated both by the results of the analyses of the physico-chemical and microbiological parameters related to the legislative standards and by the values of the similar parameters analyzed for the bottled water.

## 1. Introduction

The human body is made up of 60% water, and if we refer to young children the percentage increases to 75%, while in the elderly it decreases to 50% [1]. The largest proportion of the human body is water, hence the growing interest of specialists in defining the importance of water in the body and the risks of dehydration [2]. People can live without food for almost a month, in the presence of a normal adipose tissue, but without water, survival is not possible for more than 3 days [3].

Minerals are found in dissociated water, as electrolytes, with a positive charge (cations) or a negative charge (anions). The main cations are calcium (Ca), magnesium (Mg), sodium (Na) and potassium (K), and the category of anions includes bicarbonate (HCO_3_), chlorine (Cl), sulfate (SO_4_) and nitrates (NO_3_). To these are added the trace elements represented by metals and metalloids with a low concentration both in water and in the human body, such as manganese (Mn), iron (Fe), fluorine (F), silicon (Si), selenium (Se), zinc (Zn), etc. [4]. All these play a decisive role in the constitution and functioning of the human body. The body provides a large part of its needs for minerals from food (vegetables and meat), due to the fact that they are found in the form of organic compounds, which are more easily assimilated by the human body than inorganic ionic compounds, as minerals in water [5]. They often grow in soils whose mineral resources have long been depleted by intensive cultivation [5]. However, experts unanimously acknowledge that the intake of minerals from mineral water is not negligible, and it comes to supplement the body’s needs, especially in cases where we do not have a very varied diet in the fruits and vegetables we eat today. They often grow in soils whose mineral resources have long been depleted by intensive cultivation [6].

Water helps to recover lost fluids through metabolism, respiration, perspiration and waste removal. It helps prevent overheating, lubricates joints and tissues, keeps skin healthy and is necessary for proper digestion. It is the perfect calorie-free drink to quench your thirst and rehydrate your body [4].

From a physico-chemical point of view, no two types of mineral water are identical. The mineralogical composition of the rocks crossed by water in its underground course, the duration of this course, the mixture with other waters as well as the presence of natural CO_2_ emissions, are just a few factors that have a decisive impact on the composition and taste characteristics of mineral water [7].

Bottled drinking water, unlike tap water, is subject to verification only for a limited number of parameters. The tap water is monitored more frequently and can be consulted publicly on the website of the Public Health Directorate or on the website of drinking water supply companies such as the Water Distribution Operator, which serves the Mureș area.

The aim of the study is to compare, in terms of the physico-chemical and microbiological parameters, different brands of bottled water vs. tap water with the aim of increasing consumer confidence in drinking water quality, which could help reduce the use of plastic packaging and increase the reduction in plastic waste as well as greenhouse gas emissions with a climate change mitigation impact and a positive effect on the environment as a whole.

## 2. Materials and Methods

This study determined the physico-chemical and microbiological parameters of the drinking water distributed in the public network in the area of operation of the Water Distribution Operator in Mureș County and a sample of bottled water existing on the market in Mureş County (mineral, spring or table) and reported the results to the Romanian Drinking Water Standard (Law 458/2002, supplemented by Law 311/2004 and Decision 1020/2005 for the approval of the technical norms for the exploitation and commercialization of natural mineral waters) [8,9,10].

The data taken from the weekly published report on the physico-chemical and microbiological parameters for the drinking water distributed by the local water operator were used for analysis (Appendix A.1, Appendix A.2).

For bottled water, 20 different brands of bottled water existing on the market in Mureş County in 2022 were purchased.

Statistical Determinations

For the data identified through the water analysis, we calculated the median, minimum and maximum for both the bottled water and drinking water from the distribution system. For both data sets, we applied normality tests [11] and validity tests. The comparison of the two sets of data (bottled water vs. drinking water from the distribution system) was made using non-parametric tests (Mann–Whitney test).

The statistical calculation was performed using GraphPrism vers. 9 and the chosen significance threshold was 0.05.

## 3. Results

### 3.1. Physico-Chemical Parameters

The values of the physico-chemical parameters of the bottled water samples, on different brands, were similar (Table 1).

The only element in which we did not have significant differences between the two types of water was the oxidability (Table 2).

For the content of minerals (anions), the statistically significant differences were identified for bicarbonates, sulphates and chlorides in the water distributed in the network and the bottled one. It can be seen that the only element in which there was no statistically significant difference was the nitrate content in the water (Table 3).

In the case of cations, we identified statistically significant differences for the following elements: hardness water, calcium and magnesium content. Ammonium was the only element analyzed in which we did not identify statistically significant differences between the main’s water and bottled water. (Table 4).

### 3.2. Microbiological Determinations for Bottled Water

The number of colonies at 22 °C in the case of 9 bottled waters exceeded the microbiological parameters, namely at the no. of colonies at 22 °C. Samples 3, 7, 8, 9, 10, 11, 15, 16 and 18 exceeded 385 and a maximum of 4000 CFU/mL. The maximum value was recorded at test no. 16: flat spring water, with 4000 CFU/mL (Table 5).

The number of colonies at 37 ° C in the case of 5 bottled waters exceeded the microbiological parameters, namely at the no. of colonies at 37 °C. Samples 7, 9, 10, 11 and 15 recorded exceedances ranging from 270 to a maximum of 2560 CFU/mL. The maximum value was recorded at test no. 10: flat mineral water, with 2560 CFU/mL (Table 5).

## 4. Discussion

In the present study, we aimed to follow only the usual drinking water contaminants and the microbiological ones, between tap and bottled water, in order to identify any differences that would justify the exclusion of tap water as drinking water.

The physico-chemical and microbiological parameters for 9 of the samples in the public water network displayed on the Water Distribution Operator in Mureș County website, in January 2022, were compared with the same parameters analyzed for 20 samples of bottled water, accessible to consumers in the same area.

The analysis of the samples was completed through the same laboratory, belonging to the Water Distribution Operator in Mureș County, and the standard was representative of the Romanian Drinking Water Standard, regulated by law [8,9,10].

All drinking water samples from the public supply network fall within the drinking water standard, unlike the 9 bottled water (Table 5) samples that do not fall within the standard, due to the microbiological component. 

The results of this study are all the more important as the legal standards for drinking water distributed in public supply networks are stricter than those for bottled water [8].

Various studies have found irregularities in certain samples of bottled water, such as the presence of traces of E. coli [12,13] or arsenic [14]. These studies could not prove that bottled water is inferior in quality to municipal water, but drew attention to the regulatory framework for bottled water, which they categorized as inadequate to ensure consumers’ purity or water safety.

Other studies drew attention to the possibility of transferring chemicals from the composition of plastic bottles into the water they contain [15]. Polyethylene terephthalate (PET, PETE or # 1), the plastic used to pack most single-use bottled water bottles, is not intended to be reused, but consumers often refill and reuse these bottles. PETs are considered more stable and less prone to such transfers than other forms of plastic, but studies suggest that di (2-ethylhexyl) phthalate (DEHP) can be released by the repeated use of PET containers, a probably human carcinogen compound that affects the endocrine system [16].

Another study found high levels of antimony in bottled water in PET bottles and found that the source of the contamination was the bottles themselves, although the concentration levels were lower than those considered safe for drinking water in the US and Canada [17]. In most of these studies, it was found that the concentration of toxins increases the longer the water stays inside the plastic bottles and the higher the storage temperature [18]. Considerable attention has also been paid to the chemical transfer problems associated with Bisphenol A (BPA) [17] and plastic microparticles [19,20].

There are studies that draw attention to the possibility that bottled water may contain in certain quantities some substances with a potentially more or less harmful effect on human health: these cannot be classified as unsuitable for consumption [21,22].

Many consumers believe that bottled water is a high quality product. In reality, countless companies simply filter municipal water and bottle it [21,23]. Increased transparency in bottled water analysis could provide greater certainty about the quality of bottled water [23,24]. The possibility of informing consumers through labeling, as it applies to food [25], would provide the opportunity to identify the consumer’s profile in relation to water consumption [26,27].

On the other hand, the consumption of oil, energy and water from the production process and the transportation of bottled water, as well as the huge amounts of waste that end up in landfills or directly in nature, through which ecosystems are directly and indirectly affected, are a reality that we must be aware of, and we must take it into account when we have the opportunity to choose between different sources of water that are safe for our health [28].

Approaching integrated water management in the urban network is an important strategy to improve water security and improve the system [29,30,31].

## 5. Conclusions

The water supplied to the network can be safely consumed. This is demonstrated both by the results of the analyses of the physico-chemical and microbiological parameters related to the legislative standards and by the values of the similar parameters analyzed for the bottled water. The analysis of the water consumption behavior in the population can complete the present study, in order to intervene to promote water consumption as more accessible and cheaper by reducing the risks of chemical and biological contaminants for humans and environmental pollutants.

## Figures and Tables

**Table 1:** Physico-chemical parameters for bottled water.

BOTTLED WATER	Turbidity, FNU	pH	Oxidisability, mg/l	Conductivity S/cm	Residue sec -180 grd, mg/l	Bicarbonates (HCO_3_), mg/l	Nitrites (NO_2_), mg/l	Nitrates (NO_3_), mg/l	Sulphates (SO_4_), mg/l	Chlorides (Cl), mg/l	Total Durity G	Calcium (Ca), mg/l	Magnesium (Mg), mg/l	Ammonium (NH_x_), mg/l
Oligomineral plain water 1	0.13	8.0	1.38	184	90	73	<0.004	2.0	7.1	0.2	20.8	68.0	39.0	0.011
Oligomineral plain water 2	0.17	7.6	1.28	90	76	29	<0.004	4.9	6.8	0.8	4.5	2.8	17.9	0.007
Oligomineral plain water 2	0.17	7.7	0.77	354	182	207	<0.004	2.1	8.8	0.9	11.4	44.0	22.1	0.015
Mineral plain water 1	0.11	7.8	3.01	511	296	317	<0.004	5.5	9.2	1.9	15.2	57.0	31.0	0.016
Mineral plain water 2	0.21	7.7	0.90	615	372	354	<0.004	5.4	4.8	13.2	37.8	204.0	40.0	0.010
Mineral plain water 3	0.10	7.6	1.09	323	170	171	<0.004	5.6	8.5	2.3	10.6	61.2	8.7	0.008
Mineral plain water 4	0.19	7.9	1.18	35	202	195	<0.004	6.1	7.1	1.0	11.5	72.0	6.2	0.014
Mineral plain water 5	0.12	7.6	1.31	606	338	285	<0.004	4.2	11.9	11.0	16.4	75.1	25.3	0.010
Mineral plain water 6	0.12	7.6	1.34	119	68	65	0.012	3.2	5.3	2.0	3.5	13.5	6.9	0.016
Mineral plain water 7	0.25	8.0	1.44	310	168	181	<0.004	3.8	7.2	0.7	9.6	53.9	8.8	0.009
Alkaline mineral water	0.11	9.1	1.09	207	142	71	<0.004	0.5	26.6	3.4	0.9	1.4	3.7	0.014
Mineral water	0.11	6.3	1.57	1146	736	683	<0.004	2.0	35.6	10.5	30.8	111.0	66.1	0.026
Imported mineral water	0.26	7.9	0.74	478	254	293	<0.004	8.8	5.9	3.6	4.8	52.0	11.9	0.010
Flat water spring 1	0.14	7.6	0.70	844	482	417	<0.004	19.9	29.4	32.4	21.4	83.9	41.7	0.021
Flat water spring 2	0.21	7.7	1.66	415	240	268	<0.004	3.6	11.0	2.0	14.2	79.7	12.8	0.012
Flat water spring 3	0.13	7.5	1.12	416	234	195	<0.004	3.9	9.8	2.4	13.5	68.7	16.5	0.011
Flat water spring 4	0.15	7.9	0.90	548	334	176	<0.004	1.9	89.0	9.1	5.7	17.7	14.1	0.010
Alkaline plain water spring 1	0.21	8.1	1.18	283	208	159	<0.004	3.6	10.0	1.5	10.1	12.5	10.1	0.012
Alkaline flat water spring 2	0.22	9.4	1.95	298	190	203	<0.004	0.6	10.5	2.6	1.3	2.8	5.5	0.042
Import table water	0.18	6.9	1.12	476	488	20	<0.004	0.4	4.8	127.0	11.5	62.4	11.8	0.102

**Table 2:** Physical and chemical parameters for main’s water vs. bottled water.

**Physico-Chemical Parameters Analyzed**		**Drinking Water from the Distribution** **System** **Median (min/max)**	**Bottled Water (PET)** **Median (min/max)**	***p***
	Turbidity	0.23 (0.2/0.35)	0.16 (0.1/0.26)	0.0002 *
	pH	7.5 (7.5/7.6)	7.7 (6.3/9.4)	0.0010 *
	Oxidability	1.49 (1.07/1.95)	1.18 (0.7/3.01)	0.1862 *
	Conductivity	196 (186/218)	384.5 (35/1146)	0.0110 *
	Dry residue -180 grd	124 (76/150)	221 (68/736)	0.0017 *

* Mann–Whitney test.

**Table 3:** Mineral content (anions) in main’s water vs. bottled water.

**Anions (mg/L)**		**Drinking Water from the Distribution System** **Median (Min/Max)**	**Bottled Water (PET)** **Median (Min/Max)**	***p***
	Bicarbonate (HCO_3_)	61 (48.8/68)	195 (20/683)	0.0003 *
	Nitrates (NO_3_)	3.55 (3.17/3.77)	3.72 (0.4/19.86)	0.5695 *
	Sulphates (SO_4_)	14.6 (11.8/15.9)	9.02 (4.81/89)	0.0112 *
	Chlorides (Cl)	18.1 (17.1/20.2)	2.35 (0.2/127)	0.0003 *

* Mann–Whitney test.

**Table 4:** Mineral content (cations) in main’s water vs. bottled water.

**Cations (mg/L)**		**Drinking Water from the Distribution System** **Median (Min/Max)**	**Bottled Water (PET)** **Median (Min/Max)**	***p***
	Total Durity G	3.7 (3.4/3.9)	11.45 (0.86/37.8)	0.001 *
	Calcium (Ca)	16.8 (15.4/18)	59.10 (1.4/204)	0.037 *
	Magnesium (Mg)	5.59 (4.8/6.65)	13.45 (3.72/66.1)	0.0002 *
	Ammonium (NH_4_)	0.013 (0.008/0.019)	0.012 (0.007/0.102)	0.972 *

* Mann–Whitney test.

**Table 5:** Physico-chemical parameters for bottled water.

BOTTLED WATER	Total Coliform Bacteria, UFC/250 ml	Escherichia Coli, UFC/250 ml	Enterococi, UFC/250 ml	Clostridium Perfringens, UFC/50 ml	Pseudomonas Aeruginosa, UFC/250 ml	Number of Colonies at 22 grd, UFC/ml	Number of Colonies at 37 grd, UFC/ml
Flat oligomineral water 1	0	0	0	0	0	0	0
Flat oligomineral water 2	0	0	0	0	0	0	0
Flat oligomineral water 2	0	0	0	0	0	118	9
Flat mineral water 1	0	0	0	0	0	0	0
Flat mineral water 2	0	0	0	0	0	0	0
Flat mineral water 3	0	0	0	0	0	0	0
Flat mineral water 4	0	0	0	0	0	2080	1120
Flat mineral water 5	0	0	0	0	0	385	18
Flat mineral water 6	0	0	0	0	0	3200	2500
Flat mineral water 7	0	0	0	0	0	2400	2560
Alkaline mineral water	0	0	0	0	0	800	528
Mineral water	0	0	0	0	0	0	0
Imported mineral water	0	0	0	0	0	4	0
Flat water spring 1	0	0	0	0	0	0	0
Flat water spring 2	0	0	0	0	0	1280	270
Flat water spring 3	0	0	0	0	0	4000	20
Flat water spring 4	0	0	0	0	0	0	0
Alkaline flat water spring 1	0	0	0	0	0	800	1
Alkaline flat water spring 2	0	0	0	0	0	0	0
Import table water	0	0	0	0	0	0	0

## Data Availability

Not applicable.

## Appendix A

### Appendix A.1. Physico-Chemical Determinations for Drinking Water Distributed by Water Distribution Operator in Mureș County

**Table A1:** Physico-chemical parameters of drinking water from the Water Distribution Operator in Mureș County network.

Collection Areas Targu Mures	Turbidity, FNU	pH	Oxidisability, mg/l	Conductivity, µS/cm	Dry Residue -180 grd, mg/l	Bicarbonates (HCO_3_), mg/l	Nitrites (NO_2_), mg/l	Nitrates (NO_3_), mg/l	Sulphates (SO_4_), mg/l	Chlorides (Cl), mg/l	Hardness G	Calcium (Ca), mg/l	Magnesium (Mg), mg/l	Ammonium (NH4), mg/l
7 Nombril	0.22	7.6	1.07	204	102	49	<0.004	3.5	14.6	18.4	3.7	16.7	5.9	0.019
Centru	0.35	7.5	1.07	188	124	61	<0.004	3.5	13.4	17.1	3.4	15.4	5.4	0.009
Cornişa	0.22	7.5	1.49	196	76	62	<0.004	3.6	13.5	18.1	3.7	16.8	5.6	0.017
Mihai Viteazu	0.22	7.5	1.20	218	138	68	<0.004	3.6	15.9	20.2	3.9	18.0	5.8	0.010
Gh. Marinescu	0.24	7.5	1.95	205	96	63	<0.004	3.4	13.6	18.9	3.9	16.6	6.7	0.008
Tudor	0.20	7.5	1.49	190	130	61	<0.004	3.2	14.6	17.9	3.4	15.4	5.1	0.018
Dâmbu Pietros	0.27	7.5	1.56	199	124	62	<0.004	3.6	15.3	17.9	3.5	17.2	4.8	0.010
Gh. Doja	0.28	7.5	1.33	186	150	58	<0.004	3.7	14.6	17.3	3.4	17.1	5.0	0.017
Unirii	0.23	7.5	1.75	188	122	58	<0.004	3.8	11.8	18.5	3.9	17.3	6.2	0.013

### Appendix A.2. Microbiological Determinations for Drinking Water Distributed by Water Distribution Operator in Mureș County

Number of Colonies at 22 °C

The total number of colonies or mesophilic germs (which grow at 22 °C), a global indicator that allows the assessment of the general microbiological load of water, was analyzed by the method of incorporating in Petri dishes successive dilutions made from water, by inserting 1 cm^3^ dilution in the culture medium. The number of colonies obtained after incubation for 48 h at 22 °C was used together with the dilution used to estimate the number of mesophilic germs per cm^3^ of collected water.

Number of Colonies at 37 °C

The total number of colonies or mesophilic germs (which grow at 37 °C), a global indicator that allows the assessment of the general microbiological load of water, was analyzed by the method of incorporating in Petri dishes the successive dilutions made from collected water, by inserting 1 cm^3^ dilution in the culture medium. The number of colonies obtained after incubation for 48 h at 37 °C was used together with the dilution used to estimate the number of mesophilic germs per cm^3^ of collected water.

## References

[1] Ruta F.D., Tarcea M. (2018). Bazele Nutritiei.

[2] Zhou S.G., Chen W. (2018). Human Body Water Composition Measurement:Methods and Clinical Application. Zhongguo Yi Xue Ke Xue Yuan Xue Bao.

[3] Institutul National De Sanatate Publica. https://insp.gov.ro/download/cnmrmc/Ghiduri/Igiena%20Mediului%20si%20Apa%20Potabila/Notiuni-generale-pentru-populatie-privind-categoriile-de-apa-imbuteliata.pdf..

[4] Mihele D. (2011). Igiena Alimentaţiei: Nutriţie, Dietoterapie si Compoziţia Alimentelor.

[5] Feru A. (2012). Ghidul Apelor Minerale Naturale.

[6] Quattrini S. (2016). Natural Mineral Waters: Chemical Characteristics and Health Effects. Clin. Cases Miner. Bone Metab..

[7] Honig V., Procházka P., Obergruber M., Roubík H. (2020). Nutrient Effect on the Taste of Mineral Waters: Evidence from Europe. Foods.

[8] CAMERA DEPUTATILOR. http://www.cdep.ro/pls/legis/legis_pck.htp_act_text?idt=37178.

[9] CAMERA DEPUTATILOR. http://www.cdep.ro/pls/legis/legis_pck.htp_act?ida=50357.

[10] CAMERA DEPUTATILOR. http://www.cdep.ro/pls/legis/legis_pck.htp_act?ida=59310.

[11] Avram C., Marusteri M. (2022). Normality assessment, few paradigms and use cases. Rev. Romana Med. Lab..

[12] Curutiu C., Iordache F., Gurban P., Lazar V., Chifiriuc M.C. (2019). Main Microbiological Pollutants of Bottled Waters and Beverages. Bottled Packaged Water.

[13] Food Safety Authority of Ireland The Consumption of Bottled Water Containing Certain Bacteria or Groups of Bacteria and the Implications for Public Health. https://www.fsai.ie/WorkArea/DownloadAsset.aspx?id=9286.

[14] Consumer Reports. https://www.consumerreports.org/water-quality/arsenic-in-some-bottled-water-brands-at-unsafe-levels/.

[15] Irina D. (2012). Determination of Phthalates from Bottled Water by GC-MS. http://aerapa.conference.ubbcluj.ro/.

[16] Westerhoff P., Prapaipong P., Shock E., Hillaireau A. (2008). Antimony leaching from polyethylene terephthalate (PET) plastic used for bottled drinking water. Water Res..

[17] Cao X.-L., Corriveau J. (2008). Survey of bisphenol A in bottled water products in Canada. Food Addit. Contam. Part B 1.

[18] Zlatanović L., van der Hoek J.P., Vreeburg J.H.G. (2017). An experimental study on the influence of water stagnation and temperature change on water quality in a full-scale domestic drinking water system. Water Res..

[19] Molaee Aghaee E., Alimohammadi M., Nabizadeh R., Jahed Khaniki G., Naseri S., Mahvi A.H., Yaghmaeian K. (2014). Effects of storage time and temperature on the antimony and some trace element release from polyethylene terephthalate (PET) into the bottled drinking water. J. Environ. Health Sci. Eng..

[20] Mason S.A., Welch V.G., Neratko J. (2018). Synthetic Polymer Contamination in Bottled Water. Front. Chem..

[21] Cidu R., Frau F., Tore P. (2011). Drinking Water Quality: Comparing Inorganic Components in Bottled Water and Italian Tap Water. J. Food Compos. Anal..

[22] Zamberlan da Silva M.E., Santana R.G., Guilhermetti M., Filho I.C., Endo E.H., Ueda-Nakamura T., Nakamura C.V., Dias Filho B.P. (2008). Comparison of the bacteriological quality of tap water and bottled mineral water. Int. J. Hyg. Environ. Health.

[23] Clean Water Action. https://www.cleanwateraction.org/2020/07/29/bottled-water-human-health-consequences-drinking-plastic..

[24] Mahajan R.K., Walia T.P., Lark B.S., Sumanjit (2006). Analysis of physical and chemical parameters of bottled drinking water. Int. J. Environ. Health Res..

[25] Avram C., Avram L., Rus V., Georgescu I.M., Ruta F., Olah P. (2022). Food Labels. Consumer Understanding of the Information Contained. A Pilot Study. Progr. Nutr..

[26] Avram C., Avram L., Olah P., Rus V., Georgescu I.M., Bucur O.M., Ruta F. (2021). Knowledge about food additives among adults—Pilot study. Progr. Nutr..

[27] Saylor A., Prokopy L.S., Amberg S. (2011). What’s wrong with the tap? Examining perceptions of tap water and bottled water at Purdue University. Environ. Manag..

[28] Jia X., Varbanov P.S., Klemeš J.J., Wan Alwi S.R. (2019). Water Availability Footprint Addressing Water Quality. Water Environ. Syst..

[29] Perera M.P., Ng A.W.M., Muthukumaran S., O’Connor J., Perera B.J.C. (2018). Investigation of water quality in combined recycled water and stormwater systems. Urban Water J..

[30] Zlatanović L., Knezev A., Van der Hoek J.P., Vreeburg J.H.G. (2018). Influence of an Extended Domestic Drinking Water System on the Drinking Water Quality. Water.

[31] Rizak S., Cunliffe D., Sinclair M., Vulcano R., Howard J., Hrudey S., Callan P. (2003). Drinking water quality management: A holistic approach. Water Sci. Technol..

